# Dental caries status of Lisu preschool children in Yunnan Province, China: a cross-sectional study

**DOI:** 10.1186/s12903-018-0708-y

**Published:** 2019-01-15

**Authors:** Shinan Zhang, Yuexiao Li, Juan Liu, Weiqi Wang, Leticia Ito, Samamtha Kar Yan Li, Yanhong Li

**Affiliations:** 1 Affiliated Stomatological Hospital of Kunming Medical University, Yunnan, China; 2 Faculty of Dentistry, The University of Hong Kong, Hong Kong, Hong Kong, Special Administrative Region of China

**Keywords:** Dental caries, Ethnic, Minority, Child, China

## Abstract

**Background:**

Dental caries is still considered a major public health concern for human beings, especially minority groups and those living in disadvantaged communities. The Lisu is a minority group in China of more than 702,000 people located primarily in Yunnan Province. The present study was aimed at studying the status of dental caries status, as well as its risk factors, among Lisu children aged 5 years in Yunnan Province, China.

**Methods:**

A multistage cluster sampling method was employed for participants’ recruitment. Two calibrated dentists carried out the clinical examination with dental mirrors and CPI probes under an LED headlight. The dental caries experience was assessed by the dmft index. Oral hygiene status was evaluated using the visible plaque index (VPI). Information on the child’s socio-demographic characteristics and oral health-related practices were collected using a parental questionnaire. A zero-inflated negative binomial regression (ZINB) was employed to analyse the associations between the dental caries status and the children’s social-demographic status and their oral health–related behaviours.

**Results:**

In all, 470 Lisu children aged 5 were invited, and 404 were examined. Their mean dmft (±SD) and caries prevalence were 5.6 ± 4.8 and 80%, respectively. Their mean VPI scores were 58% ± 21%. Lisu children who brushed their teeth at least once daily had higher dmft scores, and children from high-income families were more likely to have dental caries.

**Conclusion:**

The prevalence of dental caries among Lisu children aged 5 years in Yunnan, China was high, and their caries status was severe, with a majority of carious teeth untreated. The dental caries experience of Lisu children aged 5 was related to their brushing frequency and families’ economic backgrounds.

## Background

Despite various evidences demonstrating that dental caries can be prevented, the problem remains a worldwide pandemic disease [1]. In 2010, 621 million children in the world suffered from untreated caries, and dental caries ranked as the 10th most prevalent health condition [2]. Furthermore, the distribution of untreated caries lesions was found to be unequal. A considerable number of studies conducted in several countries showed that ethnic minority people who live in underdeveloped areas shoulder a disproportionate burden of dental caries [3]. Though socio-economic status was considered a risk factor for the high prevalence of dental caries among ethnicity minority populations, underlying cultural beliefs and practices are possible mediators of the influence on oral health status through the use of homecare remedies, food preferences and dental care-seeking behaviours [4]. Untreated tooth decay can cause difficulties in eating and sleeping and can affect children’s growth; in addition, it is the main cause of absence from school [5]. Thus, disparities in the prevalence of dental caries may contribute to a broad range of socioeconomic inequalities in children’s health and academic success [6, 7].

China has a territory of 9,600,000 km^2^ with a population of 1.37 billion people. There are 55 officially recognized ethnic minority groups within China in addition to the predominant Han ethnic group. In 2010, the remaining ethnic minority people numbered nearly 113 million [4]. Ethnic groups usually have their own languages, cultures and religions and are widely distributed across China. However, many of them live in the underdeveloped hilly areas of western China [4]. Based on the results of the latest national oral health survey, the prevalence of dental caries among the Chinese children aged 5 was 71%; this percentage was approximately 5% higher than the proportion reported in 2005 [8]. However, the national oral health survey documented the dental caries status according to the province (a geographical administration division) but not ethnicity.

The Lisu people constitute the 20th largest ethnic minority group in China with a total population of more than 702,000 [4, 9]. They speak the Lisu language, which is one branch of the Sino-Tibetan family and has two scripts. Ninety-five per cent of them live in Yunnan Province, which borders on three countries: Laos and Vietnam in the south and Myanmar in the west. The majority of Lisu people live in the western mountainous areas of Yunnan Province, close to the Nu, Jinsha and Lancang Rivers. The climates vary and include tropical, temperate and frigid climates. In addition, Lisu people live in other Asian countries, such as Myanmar, Thailand and India [10].

The earliest historical record of Lisu people can be traced back to the Tang dynasty (AD 863) [11]. After the establishment of the People’s Republic of China, the Lisu’s social system developed directly from a primitive society into a socialist society. Although the Chinese government continuously invests in promoting the Lisu community’s development in terms of infrastructure, agriculture, education and health services, this population is still underprivileged. According to the available information, in 2012, around 90% of the Lisu inhabitants lived in poverty with annual incomes below the national poverty level ($170 per capita per year) set in 2009, and their main sources of income came from agriculture [11]. Starting in the late 1980s, China implemented a nine-year compulsory education law-from primary through secondary education. But on average, most of the Lisu people received 6.5 years of education [11, 12]. Some of the Lisu people believe that things in the world have spiritual power, and they perform sacrifices to relieve their pain or suffering. Maize, buckwheat and rice are their main staple foods [13]. They prefer fried meat, and lacquer seed oil is an important ingredient for dish preparation. Drinking home-brewed wine and boiled tea with castor seeds are common among their pastimes [14, 15]. These beliefs or habits may significantly affect their dental health status.

Epidemiological data for dental diseases can serve as a basis for planning and implementing services to enhance the oral health status of the Lisu children. Only one study, published in 2009, reported their dental caries status; however, that study did not follow the dental caries diagnostic criteria of the World Health Organization (WHO) and failed to study the impact of social determinants on children’s dental caries status [16]. The purpose of the present study was to study the dental caries status and its risk indicators among Lisu children aged 5 in Yunnan, China.

## Methods

### Sample size calculation and sample selection

The research protocol and related documents of this study, such as questionnaires and consent forms, were reviewed and approved by the Kunming Medical University Institutional Review Board. After obtaining the ethics approval, we performed the study in 2016 supported by the Education Bureau of Yunnan Province.

According to the results of a previous study, the prevalence of dental caries among Lisu children was estimated to be 51% [16]. With the estimated 7% width of the 95% confidence interval, approximately 400 children were required according to the sample size calculation formula (*n* = 4 × 1.962 × p × [1-p]/L^2^; [n: number of participants, p: prevalence of diseases, and L: width of 95% confidence interval]). The response rate was set to be 90%; thus, at least 450 children had to be recruited.

A multistage cluster sampling method was employed in the child recruitment process. Yunnan Province consists of 16 counties. Regarding the Lisu population distribution, most of them live in the western districts and some are scattered in the eastern areas of Yunnan Province. The ratio of the Lisu population distribution is around 8:1 in western and eastern areas, respectively [9]. A list of kindergartens in each cluster (district) was obtained from the Yunnan Education Bureau. The kindergartens were numbered and randomly selected. Among the selected kindergartens, all Lisu children aged 5 were invited up to the required number of participants in each cluster. Parental informed consent forms were collected before the fieldwork. Children in good general health status were invited, but those with systemic conditions (e.g., epilepsy, or systemic lupus erythematous), and those took long-term medications or who could not cooperate were excluded from this survey.

### Questionnaire survey

Parents or guardians who brought their children to school were invited to complete a questionnaire on the day before the clinical examination. Teachers trained by a research assistant were responsible for distributing and collecting the questionnaires. The parental responses were checked and followed up by the research assistant by phone if required. This questionnaire was derived from a previous epidemiology study and included two parts [17]:

(i) the child’s background information: gender, parental education levels and family monthly income; and
(ii) the child’s oral health-related behaviours: tooth brushing practices, snacking behaviours and dental attendance experience.

### Clinical examination

In the kindergartens, two trained and calibrated public health dentists examined the oral health status of the recruited children using CPI probes and dental mirrors under an LED headlight. The criteria for diagnosing dental caries followed the recommendations from the WHO [18]. The caries experiences of deciduous dentition were assessed using the dmft index, which involves decayed teeth (dt), missing teeth (mt), and filled teeth (ft). dt was diagnosed when a lesion was observed beyond doubt in a pit or fissure, or on a smooth surface. mt was recorded when a tooth was missing because of caries. Lastly, ft. was diagnosed if a dental filling was found on a tooth with no secondary decay. The visible plaque index (VPI) [19] was employed to evaluate the children’s oral hygiene status. The occurrence of clearly visible plaque on the labial or buccal surfaces of six index deciduous teeth (55, 53, 51, 71, 73, and 75) were scored. The proportion of the index teeth with the presence of dental plaque, which varied from 0 to 100%, was calculated. The overall consistency level of the two examiners’ assessments was 90%. Approximately 10% of participants were reassessed to assess the intra-examiner reliability, and the Kappa statistic was measured.

### Data entry and analysis

IBM SPSS Statistics version 22.0 (IBM Corp., Armonk, New York, United States) and STATA version 14 (Stata Corp., College Station, Texas, United States) were used for the data analysis. Statistically significant differences in the prevalence of dental caries between groups was detected using a chi-square test. A Mann-Whitney U test or Kruskal-Wallis H test was employed for the comparison of two or more groups of studied variables in the dmft scores. In order to explore the associations between dental caries status (dmft scores) and the socio-demographic and oral health-related behavioural determinates, the Poisson model, zero-inflated model, negative binomial model and zero-inflated negative binomial (ZINB) regression model were all used. Backward stepwise selection was employed to remove the least useful variables in each model until all the remaining variables had a statistically significant value. Lastly, the most appropriate model was selected by using the Vuong’s test. The variable of monthly family income was divided into three sub-categories, including low family incomes (less than 500RMB), middle family incomes (501-3000RMB) and high family incomes (more than 3001RMB).

## Results

From 11 kindergartens, 470 Lisu children aged 5 participated in the study, and 404 of them completed the clinical examination. The response rate was 86% (404/470). Among the non-respondents, 23 children did not cooperate in the examination and 43 of them were absent during the fieldwork. Three hundred and fifty-eight participants were recruited from western Yunnan and 46 from the east. The ratio of participants in the west and east was 8:1 which was the same as the Lisu population ratio in Yunnan Province. The results of Kappa for dmft and VPI were 0.97 and 0.88, respectively.

**Fig. 1:**
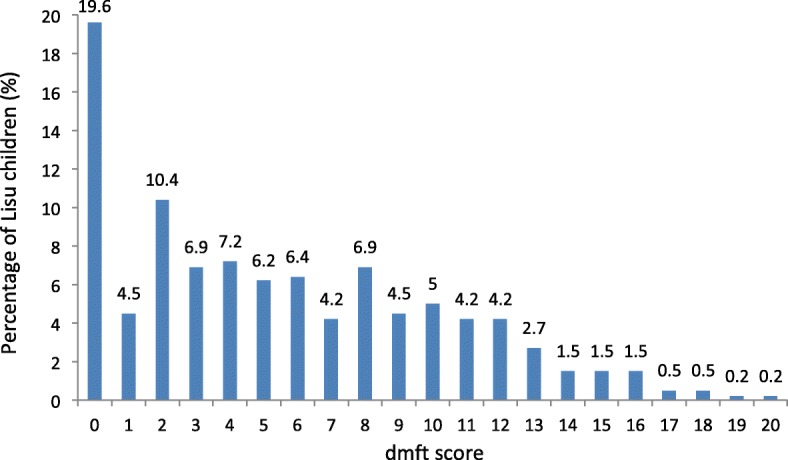
Distribution of the surveyed Lisu children according to their dmft scores

As shown in Table 1, there were 324 (80%) children with caries experience (dmft > 0). Boys accounted for 55% (223) of the surveyed children. The mean dmft score of the participants (±SD) was 5.6 ± 4.8. The dental caries experience (dmft scores) between boys and girls had no statistical differences. Of the children, 20% had more than 10 teeth with caries experiences (dmft score > 10). A majority of decayed teeth (99%) had gone untreated (mean dt = 5.6 ± 4.8) and the total mean ft. score of the participants was 0.02 ± 0.24. For boys and girls, no statistically significant differences were detected in the prevalence of dental caries (dmft> 0) or in the rank of the median dmft score (*p* > 0.05). In Fig. 1, the dmft score was a positively skewed distribution. There were no significant differences in the prevalence of dental caries between the maxillary teeth and their mandibular counterparts. However, the dental caries on the maxillary teeth were more likely to be left untreated than on the mandibular teeth (75 and 64%, respectively, *p* < 0.01), and on the posterior teeth than on the front teeth (73 and 62%, respectively, *p* < 0.01). The percentage of caries on the maxillary anterior teeth was 61%, which was correlated with that of the caries in posterior teeth (*p* < 0.001). The mean VPI score (±SD) was 58% ± 21%. Dental plaque was found on most of the surveyed children (99%, *n* = 397). Six per cent of the participants (*n* = 26) had VPI scores on all the six index teeth.

**Table 1:** Dental caries status of Lisu children

Variables (n)	Caries Prevalence	*p*-value	Mean dmft (±SD)	Mean dt (±SD)	Mean mt (±SD)	Mean ft. (±SD)	Rank of median dmft score	*p*-value
Gender		0.546						0.877
Boys (223)	79%		5.6(±4.9)	5.6(±4.8)	0.03(±0.20)	0.12(±0.01)	202	
Girls (181)	82%		5.6(±4.7)	5.6(±4.7)	0.02(±0.16)	0.33(±0.02)	203	
Total (404)	80%		5.6(±4.8)	5.6(±4.8)	0.02(±0.18)	0.02(±0.24)

Sixty-three per cent of parents reported that their children had sugary snacks less than once a day (Table 2). Of the children, two-thirds (74%) began to brush their teeth at or after 24 months. A large proportion of the participants (74%) brushed their teeth more than once a day. A small number of children used fluoride toothpaste (20%). In addition, many children (80%) had not visited a dentist within the previous 12 months.

**Table 2:** Dental caries prevalence (dmft > 0) an§d independent variables

Independent variables (%, n)	Dental caries prevalence	*p*-value	Pairwise Comparison
Background information			
Gender			
Boys (55%, 223)	79%	0.546	
Girls (45%, 181)	82%		
Father’s education			
Below secondary (54%, 219)	79%	0.860	
Secondary (20%, 81)	82%		
Tertiary or above (26%, 104)	82%		
Mather’s education			
Below secondary (54%, 207)	54%	0.253	
Secondary (20%, 81)	20%		
Tertiary or above (27%, 107)	27%		
Family monthly income			
≤500RMB (16%, 63)^(a)^	68%	0.002	(a) = (b) < (c)
501RMB-3000RMB (40%, 163)^(b)^	77%		
≥3001RMB (44%, 178)^(c)^	88%		
Oral health-related behaviours			
Frequency of sugary snack intake (times per day)			
< 1 (63%, 256)	79%	0.444	
≥ 1 (37%, 148)	82%		
Tooth brushing started age (month)			
< 24 (26%, 103)	78%	0.428	
≥ 24 (74%, 299)	81%		
Brushing frequency (times/day)			
< 1 (26%, 106)	72%	0.009	
≥ 1 (74%, 297)	84%		
Brushing with fluoride toothpaste			
Yes (20%, 71)	82%	0.939	
No (80%, 283)	80%		
Visited a dentist within last year			
Yes (20%, 79)	85%	0.275	
No (80%, 325)	79%		

The analysis of bivariate data revealed boys and girls had no statistically significant differences in the prevalence of dental caries (*p* = 0.546; Table 2). More than half of the parents (54%) had received an education below the secondary level. Parental education levels were not associated with the prevalence of the children’s caries (*p* > 0.05). A higher prevalence of caries was detected in children who brushed their teeth at least once daily (*p* = 0.009), as well as children whose families had incomes higher than 3001RMB per month (*p* = 0.002). Participants who brushed their teeth at least once daily and those from high-income families also had a higher rank of median dmft scores (Table 3). The results of Vuong’s test indicated that the zero-inflated negative binomial (ZINB) model can better predict values close to the observed data comparing with other models (*p* < 0.001). In Table 4, the dmft score was related to frequent tooth brushing (IRR = 1.363). In the zero-inflated part, children from high-income families were less likely to have ‘no caries experiences’ (OR = 0.266).

**Table 3:** Median dmft scores and independent variables

Independent variables (%, n)	Rank of median dmft score	*p*-value	Pairwise Comparison
Background information			
Gender			
Boys (55%, 223)	202	0.877	
Girls (45%, 181)	203		
Father’s education			
Below secondary (54%, 219)	197	0.245	
Secondary (20%, 81)	196		
Tertiary or above (26%, 104)	219		
Mather’s education			
Below secondary (54%, 207)	198	0.048	
Secondary (20%, 81)	185		
Tertiary or above (27%, 107)	225		
Family monthly income			
≤500RMB (16%, 63)^(a)^	179	0.003	(a) = (b) < (c)
501RMB-3000RMB (40%, 163)^(b)^	188		
≥3001RMB (44%, 178)^(c)^	224		
Oral health-related behaviours			
Frequency of sugary snack intake (times per day)			
< 1 (63%, 256)	198	0.265	
≥ 1 (37%, 148)	217		
Tooth brushing started age (month)			
< 24 (26%, 103)	184	0.068	
≥ 24 (74%, 299)	207		
Brushing frequency (times/day)			
< 1 (26%, 106)	184	< 0.001	
≥ 1 (74%, 297)	208		
Brushing with fluoride toothpaste			
Yes (20%, 71)	167	0.393	
No (80%, 283)	179		
Visited a dentist within last year			
Yes (20%, 79)	218	0.190	
No (80%, 325)	198

**Table 4:** Caries risk factors of the 5-year-old Lisu children (Zero-inflated-model, *n* = 404)

Negative binomial portion (dmft> 0)	Variables	IRR	95% C.I.	*p*-value
	Brushing frequency (times/day)			0.001
	≥1	1.363	1.136–1.637	
	< 1*			
Zero-inflated portion (dmft = 0)	Variables	OR	95% C.I.	*p*-value
	Family monthly income			0.004
	≥3001RMB	0.266	0.119–0.593	0.001
	501RMB-3000RMB	0.615	0.304–1.242	0.175
	≤500RMB*			

*Reference group

## Discussion

Due to the uneven demographic distribution of ethnic minority groups in China, it can be logistically and methodologically challenging to conduct epidemiological studies for ethnic minority groups. Different ethnic groups have their own beliefs and lifestyles, which can significantly affect dental caries status among children. Currently, there were no studies that provided updated information on Lisu children’s dental caries status, which is fundamental for properly planning and implementing community programmes among Lisu ethnic minority communities.

Although a great number of the Lisu people live in Yunnan Province, the population distribution is widely distributed across this province. Thus, the present study used a multistage sampling method to recruit Lisu preschool children from western and eastern districts, according to their population ratio in Yunnan. This was a convenient and effective sampling method for obtaining samples that represented the general population. Moreover, in mainland China, preschool education is not compulsory. Some children from disadvantaged families cannot attend kindergartens and were not included in this study. Even though the number was expected to be small, it could have an impact on the estimate of the caries status of Lisu children. In addition, regarding oral hygiene status, the plaque on the lingual surfaces of the index teeth was not recorded; thus, we might have underestimated the true VPI values, so the results of this study should be interpreted with caution.

Comparing with the goal described in the Oral Health Goals in 2020 established by the WHO that 50% of children aged 5 should be caries-free, the prevalence of dental caries among Lisu children was high [20]. The severe caries status of Lisu children may be due to the growing intake of cariogenic food but inadequate exposure to fluoride. Nevertheless, the prevalence of dental caries among Lisu children was similar to that of Dai and Bulang preschool children in Yunnan [21, 22]. Moreover, the prevalence of dental caries in Lisu children was also similar to that of nearby developing South East Asian countries, such as Myanmar (75%), Laos (89%), and Vietnam (63 to 95%) [23]. The high prevalence of dental caries in these areas might be due to limited medical resources. This prevalent caries status was also observed when compared with the lower prevalence of caries among Han children (the predominant ethnic group in China) living in prosperous areas, such as Sichuan Province (63%), Shanghai City (66%), and Beijing City (60%) [24–26]. Many Lisu children (80%) had not visited a dentist within the last year. Low awareness concerning children’s dental health among the caregivers might be one of the reasons. A substantial portion of decayed teeth (99%) were left untreated among Lisu children. This portion of untreated caries was remarkably higher than that of some developed countries. For example, in Italy, only 22% of the dental caries were untreated [27]. Socioeconomic status and oral health care systems might account for such differences in the prevalence of the untreated caries between Italian and Lisu communities. In Yunnan Province, the overall ratio of dentists to the population was 1:22324; this shortage of dental personnel was expected to be even more severe in the underprivileged Lisu communities [28]. In addition, most Lisu people need to pay out of pocket for dental treatment [11, 29], which would further limit their access to dental services. Although the number of oral health promotion programmes, such as the Comprehensive Intervention on Children’s Oral Diseases in the Central and Western Regions Project and the Oral Health Promotion and Oral Medicine Development West Action Plan, has increased in the last decade in the underdeveloped areas of China [30], the implementation of these programmes has provided no significant benefit to the dental health of this population.

In addition, because the distribution of the dmft index was not normal, with excessive zeros (dmft = 0), four commonly recommended statistical models were employed to investigate the effect of the studied independent variables on the dmft score. The Vuong’s test illustrated that the ZINB regression model had a better fit for the present data than the other models. This model included two parts: a negative binomial counts model, which explored the relationship between the studied variables and the over-dispersed count variable (dmft > 0). The other part was the logit model, which predicted the relationship between the studied variables and the excess zeros (dmft = 0).

The present study found no significant difference in the prevalence of dental caries prevalence among Lisu boys and girls, which was consistent with many previous studies [4]. In addition, though many previous studies reported a positive relationship between the frequency of consuming sugary snacks and dental caries [31], this association was not statistically significant in the present study. It should be noted that the question related to snacking habits was straightforward but too general. For instance, more detailed information on the types of sugars and the timing of sugars intake should be further investigated to better understand sugar’s influence for the dental caries status. In this study, children from high-income families were more likely to have dental caries than their peers. This finding was different from those of previous studies, which indicated that low-income families experienced more dental caries [32]. One explanation for this counterintuitive finding is that higher incomes may be associated with parental labour migration. Since the modernization of China, many adults in rural areas have immigrated to urban cities, and they usually receive higher incomes than their peers working in rural areas. Children from these families usually remain in rural regions under the care of their grandparents, single parents or other relatives [33]. Unfortunately, we failed to include information on Lisu children’s main caretakers. Thus, it is not known whether changes in caregivers in underdeveloped areas contribute to this difference in Lisu children’s dental caries experience.

In addition, it is also interesting to note that Lisu children who brushed their teeth one or more times a day were more likely to have higher dmft scores. This finding contradicts the results of various studies that involved different child populations [34]. One explanation is that when Lisu children had dental pain or discomfort, many parents forced their children to brush their teeth thoroughly as a last resort because of the limited access to dental health care. The preventive effect of fluoride toothpaste on caries is well known [34], but its effect on established caries is limited. In addition, only 20% of the parents reported the usage of fluoride toothpaste in their children’s tooth brushing. This is probably due to a lack of knowledge among the Lisu parents about fluoride for preventing dental caries and the low price of domestically produced non-fluoride toothpaste [35].

Improvements in oral health status were established as limited when methods to raise oral health knowledge and awareness were applied [36]. Thus, a community-based water fluoridation programme or a simple self-care programme via tooth brushing with affordable fluoridated toothpaste, along with parental oral health education can be options. For established severe dental caries, effective, low-cost and technically insensitive approaches, such as atraumatic restorative treatment and sodium diamine fluoride application, should be considered [37].

## Conclusion

The prevalence of dental caries among Lisu children aged 5 years in Yunnan, China was high, and their caries status was severe, with a majority of carious teeth untreated. The dental caries experience of Lisu children aged 5 was related to their brushing frequency and families’ economic backgrounds.
